# Percutaneous coronary intervention for chronic total occlusion of the left circumflex branch in mirror dextrocardia: a case report

**DOI:** 10.1186/s13256-023-04196-2

**Published:** 2023-11-22

**Authors:** Du Hailin, Qiao Hongtu, Zhang Wenyong

**Affiliations:** Cardiovascular Department, Chengdu Qingbaijiang District People’s Hospital, No.15, Fenghuang East 4th Road, Qingbaijiang District, Chengdu, 610399 Sichuan China

**Keywords:** Mirror dextrocardia, Chronic total occlusion, Percutaneous coronary intervention

## Abstract

**Background:**

Mirror dextrocardia (MDC) is a condition in which the heart is located in a mirror-image position on the right side of the chest compared to the normal position in individuals with physiological variations. Patients with MDC and chronic total occlusion (CTO) of the left circumflex branch (LCX) are extremely rare in clinical practice. The treatment of MDC-CTO-LCX differs significantly from patients without mirror dextrocardia and the same condition in terms of instrument selection and procedural techniques. In this article, we report a successful case of interventional treatment in a patient with MDC-CTO-LCX. We summarize the anatomical and electrocardiographic variations in patients with MDC-CTO-LCX, and discuss the selection of interventional instruments and techniques that can be useful for interventionists as well as the diagnostic and therapeutic considerations that can be helpful for clinical physicians.

**Case presentation:**

A male Han Chinese patient, 51, was admitted, presenting recurrent chest pain for a year and recent onset of exertional fatigue over the past week.He reported episodes of chest pain following physical activities over the past year, lasting between 5 and 20 min.Despite these symptoms, the patient did not seek immediate medical attention, and the occurrence of his chest pain has progressively lessened within the year.A week prior, the patient developed exertional dyspnea after brief walks, though without any episodes of nocturnal paroxysmal dyspnea.Upon arrival at our hospital for evaluation, he was initially diagnosed with chronic coronary syndrome, previous inferior myocardial infarction, atrial arrhythmia, and classified under the New York Heart Association functional class III.Following his admission, a chest X-ray and coronary angiography were conducted.The results indicated mirror dextrocardia and total occlusion of the left circumflex branch. Percutaneous coronary intervention (PCI) was performed on the left circumflex branch. Subsequent angiography demonstrated optimal stent positioning without evidence of hematoma or dissection.Following the procedure, the patient's symptoms of chest pain and exertional dyspnea were resolved, which led to his subsequent discharge.A follow-up electrocardiogram, 10 months post-procedure, displayed a slow and regular atrial rhythm.

**Conclusions:**

The incidence of dextrocardia is very low, and it may appear normal on an electrocardiogram; however, careful diagnosis is required when there is an abnormal direction of the *P* wave in limb leads. During the operation for chronic occlusive lesions of the right-sided coronary artery, the anomalous anatomical structure necessitates specific requirements for instrument selection and operative techniques. After revascularization of chronic occlusive vessels in dextrocardia, routine electrocardiographic examination may show false normalization, requiring caution in interpretation.

## Background

Mirror dextrocardia(MDC)with situs inversus refers to a congenital cardiac malformation where the heart is positioned opposite to its normal orientation during embryonic development. Its incidence is approximately 1 in 10,000 and is relatively uncommon in clinical practice [1, 2]. The occurrence of concomitant chronic total occlusion of the left circumflex branch (CTO-LCX) is even rarer. Due to the tortuous nature of the lesions in the left circumflex artery, the success rate of percutaneous coronary intervention (PCI) is lower [3, 4]. This article aims to enhance the understanding and diagnosis of mirror dextrocardia and chronic total occlusion of the left circumflex branch(MDC-CTO-LCX) among cardiovascular clinicians through a case analysis and literature review. The clinical characteristics and management strategies of this condition will be explored.

## Case presentation

**Fig. 1:**
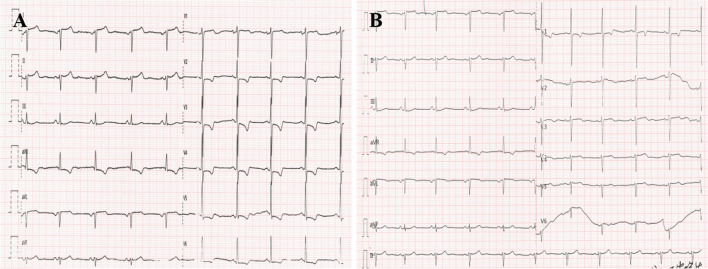
Electrocardiogram of the patient at the time of admission. **A** Preoperative normal lead placement electrocardiogram: Sinus bradycardia, inverted *P* waves in leads I and aVL, negative QRS complex; lead III shows a QS pattern with low-amplitude *T* waves, inverted *T* waves in lead aVR; lead aVF has a normal waveform, reciprocal changes between leads II and III and between leads aVR and aVL; leads V1 to V5 show gradually increasing *R* wave amplitudes, ST segment depression with a downsloping shape in V1 to V5, and biphasic *T* waves. **B** Postoperative electrocardiogram with reversed lead placement of the left and right hands: Sinus bradycardia, upright *P* waves in leads I and aVL, positive QRS complex, inverted P waves in lead aVR; lead III shows an upright *T* wave with an rS pattern; leads V1 to V4 show gradually increasing *R* wave amplitudes, ST segment depression with a downsloping shape in V1 to V5, and biphasic *T* waves

**Fig. 2:**
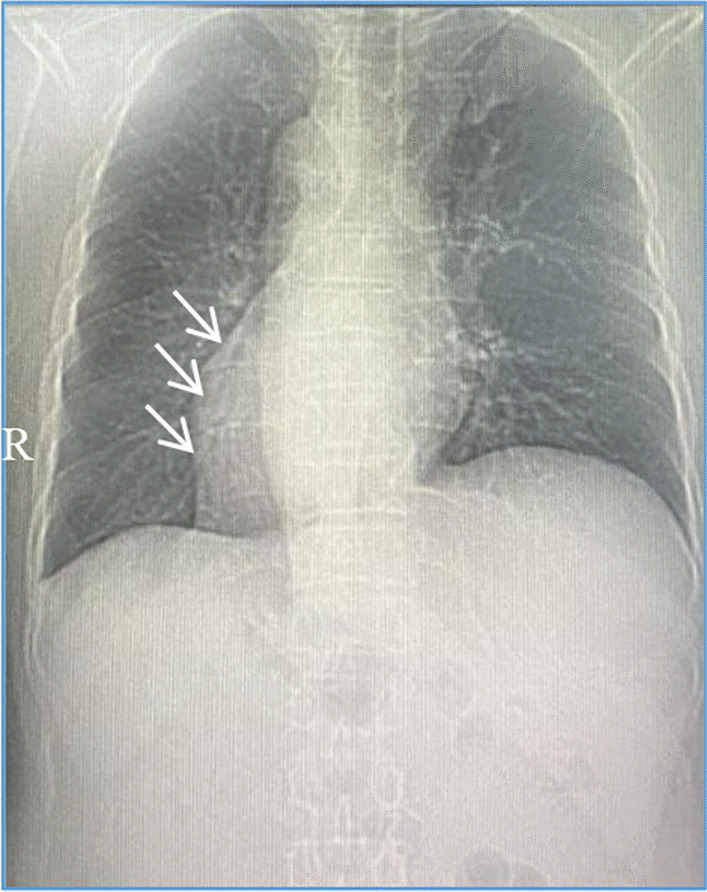
Preoperative digital radiography (DR): Demonstrates situs inversus. The position pointed to by the arrow is the left margin of the heart

**Fig. 3:**
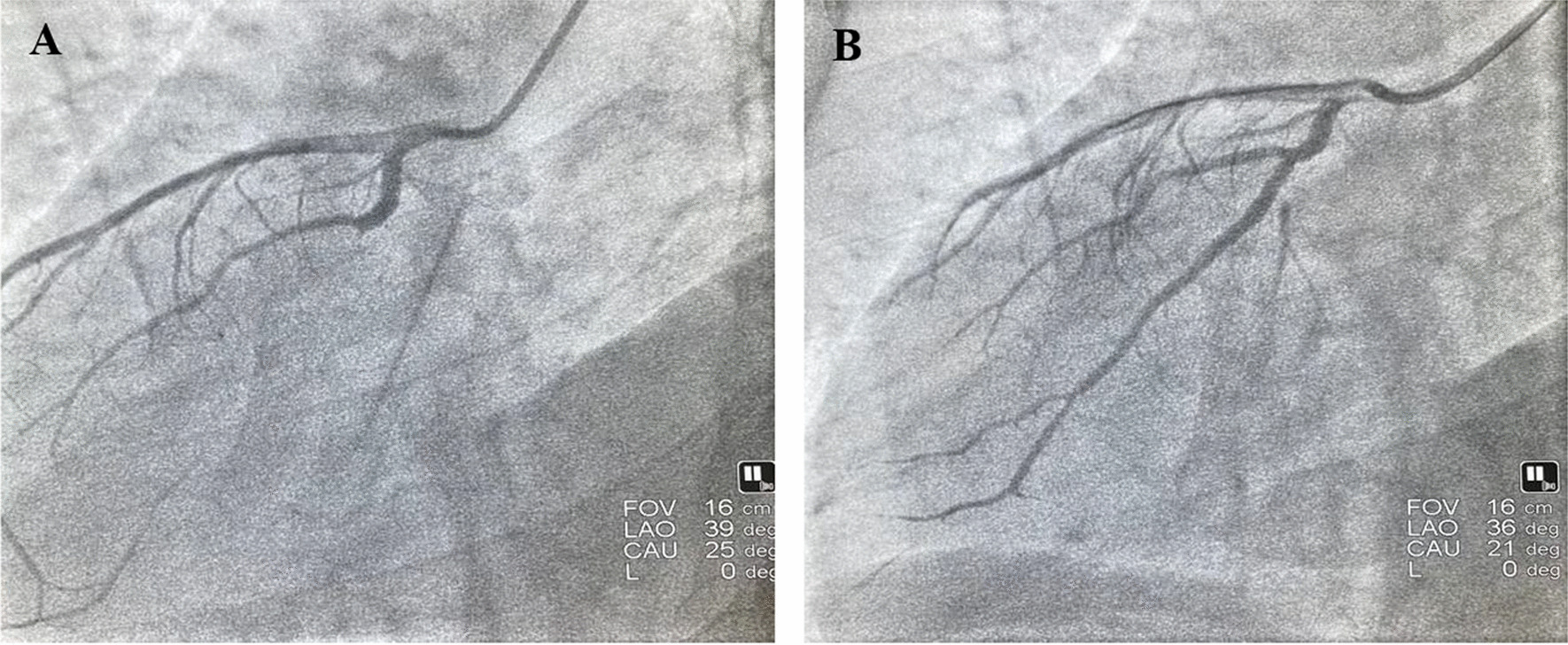
Coronary angiogram before and after percutaneous coronary intervention. **A** left circumflex branch with occlusion at the distal blunt margin of the branch. **B** Postoperative recanalization of the occluded segment of the left circumflex branch

**Fig. 4:**
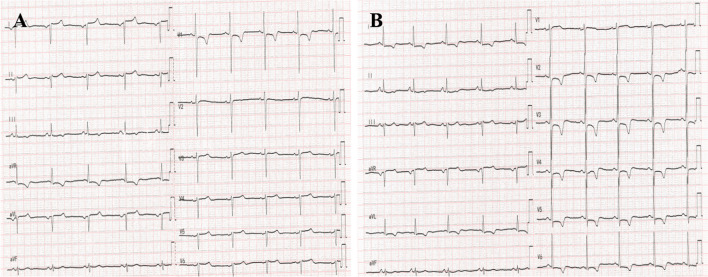
Electrocardiogram of the patient 10 months after follow-up. **A** Postoperative 10-month follow-up normal lead placement electrocardiogram: Sinus bradycardia, inverted *P* wave in lead I and aVL, negative QRS complex; lead III shows a low and wide *T* wave, upright *T* wave in lead aVR; lead aVF has a similar pattern to normal, leads II and III, aVR and aVL are reversed; leads V1 to V6 show gradually decreasing *R* wave amplitudes, and ST-T segment shows no changes. **B** Postoperative 10-month follow-up electrocardiogram with left and right hand reversal: Sinus rhythm, upright P wave in lead I and aVL, positive QRS complex, inverted *P* wave in lead aVR; lead III shows an upright *T* wave; leads V1 to V6 show gradually increasing *R* wave amplitudes, ST segment is sloping downwards, and *T* wave is biphasic

The patient was a 51-year-old male Han Chinese who was admitted to the hospital due to recurrent chest pain for a year, along with fatigue after exertion over the past week. In the year preceding admission, the patient experienced recurrent chest pain following physical activity, with variable duration of 5–20 min, but did not pay much attention to it. The frequency of chest pain episodes had gradually decreased in the past year. One week ago, the patient presented with exertional dyspnea, experiencing difficulty in breathing after walking only a block, with no incidents of nocturnal paroxysmal dyspnea. The patient subsequently sought medical consultation at our hospital. The patient denied any history of hypertension or diabetes but had a long-term smoking habit. The electrocardiogram (ECG) showed sinus bradycardia, with inverted P waves in leads I and aVL, and negative QRS complexes. Lead III exhibited a QS pattern with low flat *T* waves, while lead aVR showed inverted T waves. Lead aVF exhibited a normal pattern, while leads II and III, and aVR and aVL, had interchanged patterns. Leads V1 to V5 showed a gradual increase in *R* wave amplitude, ST segment depression with a downsloping shape in leads V1–V5, and biphasic T waves (Fig. 1A, B). Rapid bedside testing of cardiac biomarkers in the emergency department revealed troponin I (TnI) levels of 0.13 ng/ml (normal reference value < 0.30 ng/ml), creatine kinase-MB (CK-MB) levels of 2.10 ng/ml (normal reference value < 5.0 ng/ml), and myoglobin (MYO) levels of 34.4 ng/ml (normal reference value < 58.0 ng/ml). Cardiac biomarkers were rechecked after 3 h, indicating high-sensitivity cardiac troponin I (hs-cTnI) levels of 14.5 ng/L (normal reference value < 30 ng/L), CK-MB mass levels of 1.4 μg/L (normal reference value 0.6–6.3 μg/L), and MYO levels of 23.7 μg/L (normal reference value 17.4–105.7 μg/L). On admission, the blood pressure was measured as 118/70 mmHg, heart rate was 62 beats per minute, and the cardiac apex was located 0.5 cm inside the right clavicular midline. No murmurs were detected in any valve area. The diagnosis upon admission was as follows: (1) Chronic coronary syndrome, old inferior myocardial infarction, atrial arrhythmia, and class III heart failure. Chest radiography performed after admission revealed dextrocardia with increased lung markings (Fig. 2). The pre-test probability of coronary heart disease in the patient was 32%. With the patient's consent, coronary angiography was performed via digital subtraction angiography (DSA) on June 6, 2022. Intraoperatively, right-sided heart positioning was observed under fluoroscopy. LAO 45° was selected, and right coronary selective angiography was performed, revealing approximately 60% stenosis in the distal segment. No calcification or stenosis was observed in the anterior descending artery in the CRA projection. The middle segment of the first diagonal branch showed 60% stenosis (Table 1). LAO 39° and CAU 25° were selected, demonstrating a chronic total occlusion of the left circumflex branch after branching from the obtuse marginal branch, with retrograde filling from the anterior descending artery. Percutaneous coronary intervention (PCI) was performed on the left circumflex branch. LAO 39° and CAU 25° were selected to fully expose the occluded segment, and a Launcher JL 6F 3.5 guiding catheter was used with good coaxiality. The Sion guidewire could not pass through the occluded segment, but with the support of the Finecross microcatheter, the Fielder XT guidewire successfully reached the distal segment. Multiple projections were obtained, and the guidewire was positioned within the true lumen. The Fielder XT guidewire was used as the working guidewire, and the Inoue semi-compliant balloon (2.015 mm) was used to dilate the occluded segment, applying a pressure of 4–10 atm. The Excrosal stent (2.2519 mm) was placed in the proximal-to-distal segment of the left circumflex branch, applying a pressure of 8 atm. The NC Sprinter balloon (2.5*12 mm) was used to dilate the stent in the distal-to-proximal segment four times, applying a pressure of 10–14 atm. (Table 2).Angiography showed good stent apposition without any evidence of hematoma or dissection (Fig. 3). Follow-up revealed no chest pain, and the patient was discharged without experiencing any respiratory difficulties during normal activities. A follow-up electrocardiogram was performed 10 months postoperatively (Fig. 4A, B).

**Table 1:** Coronary angiography position and degree of stenosis

Coronary artery location	Observation position	Degree of stenosis
Left main trunk	RAO 5° + CAU 30°	No stenosis
Left anterior descending branch—first diagonal branch	RAO 5° + CRA 36°	60%
Left circumflex branch (culprit vessel)	LAO 39° + CAU 25°	100%
Right coronary artery	LAO 45°	60%

RAO: Right Anterior Oblique, CAU: Caudal, CRA: Cranial, LAO: Left Anterior Oblique

**Table 2:** Relevant parameters and equipment during percutaneous coronary intervention procedure

Surgical approach	Working position	Guiding catheter	Guidewire	Microcatheter	Compliant balloon	Stent type	Non-compliant balloon
Right radial artery	LAO36° + CAU 21°	Launcher 6F JL 3.5	Fielder XT	TERUMO Finecross	YINYI 2.0*15 mm	Excrossal 2.25*19 mm	Medtronic NC Sprinter 2.5*12 mm

LAO: Left Anterior Oblique, CAU: Caudal, JL: Judkins Left

## Discussion

Research has shown that due to anatomical differences, CTO-LCX is more tortuous, resulting in a lower success rate of percutaneous coronary intervention (PCI) compared to CTO of the left anterior descending artery (LAD) and right coronary artery (RCA) (LCX 84.6%, RCA 91.7%, LAD 94.7%) [3]. The combination of MDC and CTO of the LCX undoubtedly increases the difficulty of the PCI procedure. Studies have demonstrated that successful recanalization of CTO in the LAD significantly reduces long-term mortality, while successful recanalization of CTO in the RCA does not lower long-term mortality rates [5–8]. Therefore, further understanding the clinical characteristics and PCI treatment methods for MDC-CTO-LCX can improve the success rate of treatment and long-term prognosis for these patients.

In this report, we present a case of CTO in a patient with dextrocardia who underwent percutaneous coronary intervention. The pre-procedure and follow-up electrocardiograms (ECGs) exhibited specific characteristics, and abnormal ECG changes or pseudonormalization can lead to misdiagnosis or missed diagnosis. Additionally, the transposition of the systemic cardiovascular system presents specific challenges during coronary intervention procedures.

The ECG manifestations of right-sided heart in this case were as follows: inverted *P* waves and negative QRS complexes in leads I and aVL; QS pattern with low flat *T* waves in lead III and inverted *T* waves in lead aVR; normal pattern in lead aVF; interchanged patterns in leads II and III, and aVR and aVL; gradual increase in R wave amplitudes in leads V1 to V5; downsloping ST segment depression in leads V1 to V5; and biphasic *T* waves. At the 10-month follow-up, the ECG in limb leads showed no significant changes, while the precordial leads displayed normal *T* waves and ST segments. However, when the left and right hands were reversed, significant *T*-wave changes were observed. Failure to recognize the ECG changes caused by visceral transposition in patients with cardiovascular disease during readmission can easily lead to missed diagnosis of anterior myocardial ischemia.

The choice of instruments such as microcatheter, guidewire, and procedural techniques can affect the success rate of PCI [9, 10]. The femoral artery approach is the classic route for PCI procedures; therefore, the currently available interventional devices are designed for femoral artery access [11]. However, in recent years, considering patient tolerance, postoperative recovery time, intraoperative observation, and protection, the radial artery approach from the right side has become popular in most centers, with the left side being chosen only when right-side access is difficult [12–14]. The main differences between the left and right radial artery approaches lie in the fact that the right side often has a sharp angle and large variations at the point where the brachiocephalic trunk joins the aortic arch, whereas the left radial artery can overcome these limitations. The procedural techniques for the left radial artery approach are similar to those for femoral artery access, but the left radial artery approach often requires a larger guiding catheter than the right side [15].

In this case of MDC-CTO-LCX, the choice of instruments and procedural techniques were as follows: due to the visceral transposition, the right radial approach was equivalent to the left radial approach in a normal situation. Considering the normal width of the patient's aortic sinus, we chose the JL 6F 3.5 guiding catheter, which provided good support during the procedure. Initially, a relatively soft working guidewire (Sion) was selected, but it could not pass through the lesion. Subsequently, the guidewire was upgraded, and with the support of the Finecross microcatheter, the Fielder XT guidewire successfully crossed the lesion. Multiple projections were used to confirm the guidewire position within the true lumen. No guidewire exchange was performed, and the subsequent procedure was completed using the XT guidewire.

## Conclusions

MDC is rare and can lead to misdiagnosis when the ECG appears normal but shows abnormal P-wave orientations in limb leads. The peculiar anatomical structure in CTO of the right-sided heart requires specific instrument selection and procedural techniques during intervention. After successful recanalization of CTO in the right-sided heart, routine ECG examinations may falsely indicate normalization, necessitating careful interpretation.

## Data Availability

Data sharing is not applicable to this article as no datasets were generated oranalyzed during the current study.
